# Interaction of Ginseng with *Ilyonectria* Root Rot Pathogens

**DOI:** 10.3390/plants11162152

**Published:** 2022-08-19

**Authors:** Isadora Bischoff Nunes, Paul H. Goodwin

**Affiliations:** School of Environmental Sciences, University of Guelph, 50 Stone Road East, Guelph, ON N1G 2W1, Canada

**Keywords:** jasmonic acid, *Panax*, resistance, salicylic acid, virulence

## Abstract

The *Ilyonectria radicicola* species complex (A.A. Hildebr.) A. Cabral and Crous 2011 contains species of soilborne necrotrophic plant pathogens. The most aggressive to ginseng roots is *I. mors-panacis*, whereas *I. robusta*, *I. crassa*, *I. panacis* and *I. radicicola* are less aggressive. Infected ginseng roots show orange-red to black-brown lesions that can expand into a severe root rot, known as disappearing root rot, where only epidermal root tissue remains. Leaves become red-brown with wilting, and stems can have vascular discoloration with black-brown lesions at the base. Less aggressive *Ilyonectria* species trigger jasmonic acid (JA)-related defenses inducing host ginsenosides, pathogenesis-related (PR) proteins, wound periderm, and cell wall thickening. In contrast, *I. mors-panacis* triggers reactive oxygen species (ROS) and salicylic acid (SA) production but suppresses JA-related defenses and ginsenoside accumulation. It is also able to suppress SA-related PR protein production. Virulence factors include potential effectors that may suppress PAMP (Pathogen Associated Molecular Patterns) triggered immunity (PTI), polyphenoloxidases, Hsp90 inhibitors, siderophores and cell-wall-degrading enzymes, such as pectinases. Overall, *I. mors-panacis* appears to be more aggressive because it can suppress JA and SA-related PTI allowing for more extensive colonization of ginseng roots. While many possible mechanisms of host resistance and pathogen virulence mechanisms have been examined, there is a need for using genetic approaches, such as RNAi silencing of genes of *Panax* or *Ilyonectria*, to determine their importance in the interaction.

## 1. Introduction

The genus *Panax* is composed of perennial herbaceous plants whose roots are harvested for medicinal applications, such as improved cognitive functions, lowered blood pressure and stabilized heart rate [1]. Asian ginseng (*Panax ginseng*), Chinese ginseng (*Panax notoginseng*) and American ginseng (*Panax quinquefolius*) are the most widely cultivated species [2]. Commercial cultivation requires intensive management and typically lasts for 3 to 4 years with the stems dying back at the end of each growing season [3]. Ginseng pathogens can cause significant losses with several producing root rots, including *Alternaria panax*, *Phytophthora cactorum*, *Sclerotinia sclerotiorum* and *Cylindrocarpon destructans* [4].

*Cylindrocarpon destructans* has been divided into *forma specialis* based on host with *C. destructans* f. sp. *panacis* having specificity to ginseng [5]. Highly aggressive strains to ginseng are *C. destructans* f. sp. *panacis*, whereas less aggressive strains are other *Cylindrocarpon* species [6]. *Cylindrocarpon*-like fungi were originally classified into five teleomorphic genera: *Ilyonectria*, *Neonectria*, *Rugonectria*, *Thelonectria* and *Campylocarpon* [7]. The *Ilyonectria radicicola* species complex was later reclassified into 14 new *Ilyonectria* species and 4 previously described ones [8]. *I. mors-panacis*, *I. crassa*, *I. robusta* and *I. panacis* were described as being isolated from ginseng species with *I. mors-panacis* matching the highly aggressive *C. destructans* f. sp. *panacis.* A later classification resulted in five species of *Ilyonectria* and named *C. destructans* as *Ilyonectria destructans* as the name *C. destructans* was generated prior to *C. radicicola* [9]. However, the classification of Cabral et al. [8] currently appears to be most used by ginseng researchers. One issue is that earlier studies only referring to *C. destructans* make it difficult to compare to more recent studies as it is unknown if the fungus was highly or weakly aggressive to ginseng.

**Figure 1:**
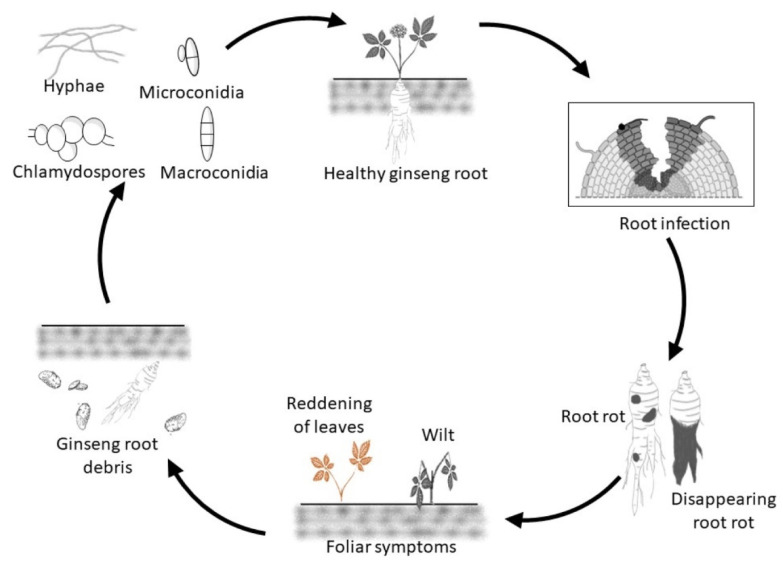
*Cylindrocarpon*/*Ilyonectria* spp. disease cycle on ginseng. Illustration created with BioRender [18].

*Cylindrocarpon*/*Ilyonectria* spp. can survive in soil in infected host debris as well as a saprophyte on dead organic matter producing mycelium and conidia, but long-term survival depends on chlamydospores that can remain viable for many years in the soil (Figure 1) [10]. The formation of chlamydospores by *Cylindrocarpon*/*Ilyonectria* spp. has been reported in vitro from conidia and hyphae [11]. The ability to thrive in low oxygen concentrations also allows *C. destructans* to grow in lower soil horizons [10].

The fungus likely detects a nearby susceptible plant by host signaling compounds exuded from the roots, such as ginsenosides [12], which triggers germination of chlamydospores, microconidia and macroconidia and then growth toward the host. Host infection normally occurs via wounds [13], usually starting from the root tips [14], but not the meristem [15]. Once it has successfully infected the host, invasion of the root occurs by hyphae growing inter- and intracellularly [16] resulting in rotting of the tissues (Figure 1). Eventually, hyphae spread to the host surface to produce micro- and macroconidia that may form chlamydospores in the soil [17].

Typical symptoms in infected ginseng roots are dry orange-red to black-brown areas with a strong odor [14,19]. The root rot can be restricted lesions resulting in plant stunting or more widespread resulting in plant death with shriveled, scaly, blackened roots where only epidermal root tissue remains, which is commonly referred as disappearing root rot (Figure 1) [20]. Symptoms in aerial parts can be barely noticeable to red-brown leaves, which can be confused with the symptoms due to *Phytophthora* and *Fusarium*, to wilting with vascular discoloration of the stem and black-brown lesions at the base of the stem, particularly when roots are highly rotted (Figure 1) [3,21]. Infection by *Cylindrocarpon*/*Ilyonectria* spp. can result in secondary invasion by non-pathogens, such as *Rhizopus*, under wet conditions [3]. Distribution and spread of the disease can be observed in the gardens as concentric areas of wilted and dead plants that eventually coalesce [22].

There are many soil fungi other than *Ilyonectria* spp. that can cause root rot of ginseng [23], making it difficult to assign losses only due to *Ilyonectria*. Although not impossible to be replanted, losses due to severe *Ilyonectria* spp. root rot can reach 100% [24]. While the cause of replant disease is not fully established, infection by *Ilyonectria* spp. have often been associated with replant disease [8,25]. Therefore, understanding the molecular and cellular basis of the host response and pathogen virulence is important for improving ginseng production, and this review will examine the relevant literature in that area.

Most of the information about the molecular/cellular interactions between *Cylindrocarpon*/*Ilyonectria* spp. and ginseng roots is based on studies of infections with restricted black-brown lesions of *P. ginseng* [26,27,28,29,30,31,32], with only a few studies of *P. quinquefolius* [20,33] and *P. notoginseng* [34]. While a few of these studies have compared host response between aggressive and less aggressive species of *Ilyonectria* [27,28,31], none have examined roots described with disappearing root rot symptoms. Thus, it is not clear if the results obtained with restricted *Ilyonectria* lesions are comparable to those with disappearing root rot, or how different are the responses of roots to isolates differing in virulence within the same species of *Ilyonectria*.

Rusty root is another ginseng disease where areas of the root periderm turn red to orange-brown [20]. While some researchers consider that *I. mors-panacis* also causes rusty root [35], others consider *I. mors-panacis* to cause only root rot while rusty root is a result of incompatible interactions between ginseng roots and the weakly aggressive *Ilyonectria* species, *I. robusta*, *I. crassa* and *I. panacis* [36]. However, there are other hypotheses, such as rusty root being related to cellulase and pectinase activities of endophytic bacteria, such as *Lysobacter gummosus* and *Pseudomonas marginalis*, as well as *C. destructans* [37]. Another hypothesis has related rusty root to nitrate-dependent iron-oxidizing bacteria, such as those in the Acidobacteria and Chloroflexi, with no association found between *Ilyonectria* species and the amount of rusty root [38]. As there are considerable differences in the interpretation about the relationship between *Ilyonectria* and rusty root disease, it will not be considered in this review.

## 2. Triggered Immunity

Plant pathogen infections trigger a wide range of changes in host gene expression. For differentially expressed genes (DEGs) in *P. ginseng* roots infected by *C. destructans*, it was shown that after infection, there were 538, 513 and 2845 DEGs up-regulated at 0.5, 4- and 12-days post-inoculation (dpi), respectively, with a peak in the level of expression at 0.5 dpi [29]. There were also 201, 69 and 280 DEGs down-regulated at 0.5, 4 and 12 dpi, respectively, with a peak at 0.5 dpi. Gene ontology of up-regulated DEGs at 0.5 dpi revealed that most of them were classified as a defense response to fungi, such as the ethylene (ET)-regulated transcription factor (TF) *ERF2*, and jasmonic acid (JA)-regulated TFs *TIFY10A*, *TIFY10B* and *MYB108*. Gene ontology of down-regulated DEGs at 0.5 dpi showed that most of them were related to basal metabolic processes, such as carbon fixation and photosynthesis, as well as a few related to resistance, such as pathogenesis-related (PR) proteins. A shift from growth, such as basal metabolic responses, to plant defenses during infection is a common observation that pathogen infections slow plant growth, thus reducing the need for assimilates, and shifts resources towards defenses [39]. The relationship of the defense response to JA was not surprising as root rot pathogens typically induce defenses related to JA rather than salicylic acid (SA) signaling [40]. It was concluded that the impact of infection on gene expression was more acute at an early stage of infection but broader at later stages. However, it is unknown if the isolate used was that of the more aggressive *I. mors-panacis* or less aggressive *I. robusta*, *I. crassa*, *I. panacis* or *I. radicicola*, as the authors only referred to the pathogen as *C. destructans*.

The same study described the host response as being related to effector triggered immunity (ETI) based on resistance (R) genes that were dramatically induced by the pathogen at 0.5 dpi [29]. ETI is characterized by a rapid and strong host response, typically a hypersensitive response that stops pathogen growth within 1–3 days [41]. However, ETI was unlikely to have occurred as there were changes in gene expression even at 12 dpi indicating that the pathogen was continuing to invade the roots. Thus, it is more likely that it was PAMP triggered immunity (PTI) with pathogen associated molecular patterns (PAMPs) triggering a strong host response at 0.5 dpi followed by a decline in gene expression due to effectors suppressing PTI. The broader host response at 12 dpi was possibly due to PTI triggered by PAMPs and damage associated molecular patterns (DAMPs) as the amount of tissue rot expanded.

RNA sequencing of *P. ginseng* roots infected with an isolate of the less aggressive *I. robusta* examined three time points: 1.5 and 3 dpi described as the initial stage of infection, and 6 dpi described as the adaptive stage of infection [42]. The largest number of up-regulated and down-regulated DEGs was 4293 and 4537, respectively, at 1.5 dpi. Most of the DEGs for cell-surface pattern-recognition receptors (PRRs), *MYB*, *WRKY* and ET-related TFs and components of PTI and ETI, such as mitogen-activated protein kinases (MAPKs), that were up-regulated at all three time points, indicating a defense response at all time points. However, there was a peak of expression at 1.5 dpi for DEGs for ET-related TFs and components of PTI and ETI, such as MAPKs, indicating that the pathogen was recognized by 1.5 dpi. At 3 dpi, there was a peak of expression for DEGs for *MYB* and *WRKY* TFs, indicating that defense signal transduction was induced slightly later.

Since most *WRKY* TFs are related to SA-regulated defence responses [43], and many *MYB* TFs are related to JA-regulated defence responses [44], this indicates that both SA and JA were involved in the host response. Most of the auxin-related DEGs were down-regulated at 1.5 dpi and slightly up-regulated at 3 and 6 dpi showing that there was early plant growth impairment that later returned to near pre-infection levels. It is well established that pathogen infections can impair plant growth as the plant shifts resources from growth to defence [39]. Similarly, most of ROS-related DEGs were also down-regulated at 1.5 dpi and slightly up-regulated at 3 and 6 dpi, which was believed to reflect a reduced defence response that later returned to near pre-infection levels. The authors concluded that the highest impact of *I. robusta* on gene expression was at the initial stage of infection, eventually returning to near pre-infection expression at the adaptive stage of infection [42].

Overall, it appears that *Cylindrocarpon*/*Ilyonectria* spp. infections can rapidly trigger a rapid host response, likely related to PTI, with later broader scale changes that perhaps are also due to PTI from damaged host cells as the pathogen continues to invade the root. Host basal metabolism and growth shifts towards plant defenses with PRRs, phosphorylation cascade, ROS and activation of TFs related to the defense hormones, SA, ET and JA. There is no clear evidence for ETI with rapid host cell death and cessation of pathogen growth, although ETI may be related to rusty root symptoms [36], as the superficial damage in rusty root could be related to the hypersensitive response.

## 3. Ginsenosides

Infections by *Cylindrocarpon*/*Ilyonectria* spp. of ginseng roots can trigger the production of ginsenosides, which are saponins with antifungal activity. Growth of *C. destructans* was significantly inhibited by 80% when protopanaxatriol (PPT) ginsenosides were added to V8 media but was significantly enhanced by almost 130% when protopanaxadiol (PPD) ginsenosides were added to the media [45]. Although growth of *I. robusta* was reduced by 20% when PPT ginsenoside was added to V8 media, there was no significant difference observed with the addition of PPD ginsenoside [27]. In contrast, growth of *I. mors-panacis* was reduced by 45% and 40% when PPT or PPD ginsenoside, respectively, were added to V8 media, and growth of *I. leucospermi* was reduced by 15% and 10% when PPT or PPD ginsenoside, respectively, was added to V8 media. Although all isolates showed growth inhibition by some form of ginsenoside, different species and perhaps even isolates can differ greatly in their sensitivity to ginsenosides. As both PPD and PPT ginsenosides accumulate in ginseng roots and can have fungitoxic activity against some *Ilyonectria* sp., ginsenoside biosynthesis during the infection process could therefore contribute to pathogen resistance.

Changes in ginsenoside root content were related to differences in aggressiveness of *Ilyonectria* sp. possibly due to triggering or suppression of resistance or the ability of the fungi to degrade ginsenosides in *P. ginseng* roots grown in pots [27]. The disease severity indices of roots infected with *I. mors-panacis* was 4.99 at 30 dpi, whereas it was 1.95 and 1.85 at 30 dpi with *I. robusta* and *I. leucospermi*, respectively, showing the difference in aggressiveness. Infection by *I. mors-panacis* decreased PPT ginsenosides in roots by 30.0% and PPD ginsenosides by 26.8% at 30 dpi, compared to the water control. However, infection by *I. robusta* and *I. leucospermi* significantly increased PPT ginsenosides by 20.0% and 10.0%, and PPD ginsenosides significantly increased by 43.9% and 46.3% at 30 dpi, respectively, compared to the water control. The decrease in ginsenoside root content was not associated with *I. mors-panacis* degrading ginsenosides as none of the isolates could degrade ginsenosides based on an in vitro fungitoxicity assay. It appeared to be due to suppression of triggering ginsenoside root content by the more aggressive isolate, which was not true for the less aggressive isolates. It appeared that *I. mors-panacis* may have been more aggressive due to effectors that were better able to suppress PTI than those of less aggressive *I. robusta* and *I. leucospermi*.

JA can increase ginsenoside root content, such as exogenous JA increasing ginsenoside content by 5-fold in ginseng roots [46]. Based on the relationship between ginsenosides and JA, the expression of several TFs potentially associated with JA and SA regulation was examined to determine if they were involved in ginsenoside biosynthesis in *P. ginseng* roots infected by *I. mors-panacis* [26]. Infection of ginseng roots by *I. mors-panacis* decreased PPT ginsenoside (Rg1 + Re + Rf) by 68.2% and PPD ginsenoside (Rb1 + Rc + Rb2 + Rd) by 70.0% at 30 dpi. At 4 dpi, only the JA/SA-regulated *PgWRKY22* was slightly induced, whereas at 8 dpi, *PgWRKY22* and the SA-regulated *PgMYC2b* were suppressed and the JA-regulated *PgMYB3* was slightly induced. At 16 dpi, *PgWRKY22*, *PgMYB3, PgMYC2b* and *PgMYC2a* were all strongly suppressed. Resistance to *I. mors-panacis* was increased by treating roots with silicon, which was associated with higher ginsenoside content. Infected silicon treated roots had a weak induction of *PgMYC2b* at 4 dpi, a strong induction of *PgMYB3* and *PgWRKY22* at 8 dpi, and a strong induction of *PgMYC2a* at 16 dpi. However, *PgMYC2b*, *PgMYB3* and *PgWRKY22* were still suppressed at 16 dpi. Thus, it appears that *I. mors-panacis* can trigger a weak early induction of two of the four TFs, but it is able to later suppress the response based on the later suppression in expression of all four TFs and ginsenoside root content, perhaps due to effector-triggered suppression of PTI. Silicon-induced resistance could result in higher expression of both SA and JA-regulated TFs and higher ginsenoside root content. The results with non-treated and silicon-treated roots show a connection between resistance to *I. mors-panacis* with defense-related TF expression and ginsenoside accumulation, indicating that ginsenosides act as defence compounds.

Based on the infection of ginseng roots by the more aggressive *I. mors-panacis* resulting in decreased root ginsenoside content [27], a follow-up study investigated SA levels and the expression of JA-, SA- and ginsenoside biosynthesis-regulated gene expression during infection by more or less aggressive *Ilyonectria* spp. in *P. ginseng* roots [26]. SA concentration was significantly higher than the control for *I. mors-panacis* at 4 hpi and was even higher at 16 dpi, but that did not occur with *I. robusta.* Total ginsenoside root content did not change significantly until 16 dpi with *I. mors-panacis* when it slightly greater than the control. By comparison, *I. robusta* infection increased total ginsenoside root content first at 4 dpi with a much greater increase compared to the control by 16 dpi. At 4 dpi with *I. mors-panacis*, there was reduced expression of the *P. ginseng* ginsenoside biosynthesis-regulated genes of farnesyl pyrophosphate synthase (*PgFPS*), squalene synthase 1 (*PgSS1*), squalene epoxidase 1 (*PgSE1*) and dammarenediol synthase (*PgDDS*), while there was increased expression of the JA synthesis gene lipoxygenase 6 (*PgLOX6*) and the SA synthesis gene phenylalanine ammonia-lyase 1 (*PgPAL1*). At 4 dpi for *I. robusta*, however, there was an increased expression of the ginsenoside biosynthesis-regulated genes and *PgPAL*, but not *PgLOX6*. At 16 dpi with *I. mors-panacis*, there was reduced expression of all the ginsenoside biosynthesis-regulated genes, but increased expression of *PgPAL* and *PgLOX6*. In contrast at 16 dpi with *I. robusta*, there was increased expression in the ginsenoside biosynthesis-regulated genes. There was also a much greater increase in *PgLOX6* expression but less of an increase in *PgPAL* expression compared to *I. mors-panacis*. Thus, there was an inverse relationship between higher aggressiveness of *I. mors-panacis* with ginsenoside and JA synthesis gene expression, whereas there was a direct relationship with SA and SA-synthesis gene expression. Down regulation of JA-regulated gene expression could be due to triggering of SA-regulated gene expression, as it is known that they are antagonistic to each other [47]. However, it is possible that the down-regulation of the JA-regulated gene expression could be due to effectors from *I. mors-panacis* that are suppressing it, as many fungal pathogen effectors are well known for suppressing PTI [48].

**Figure 2:**
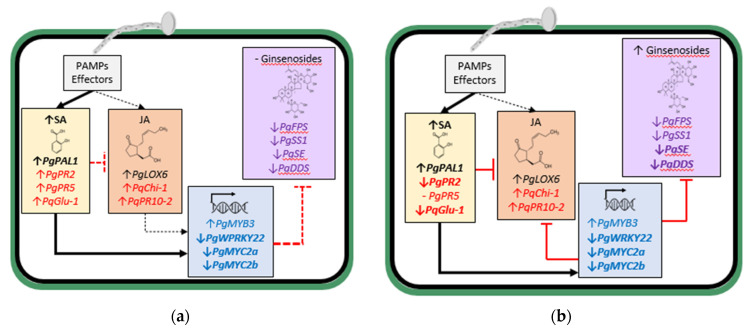

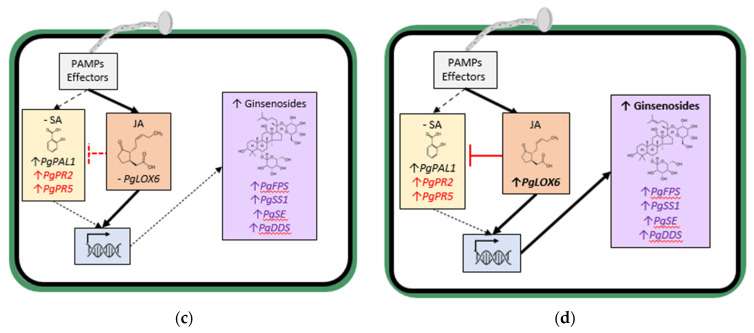
Aspects of SA and JA-associated host responses in ginseng roots with (**a**) the more aggressive *I. mors-panacis* at the early stage (0.5–8 dpi); (**b**) and late stage of infection (9–30 dpi); (**c**), and the less aggressive *Ilyonectria* spp. at the early stage (0.5–8 dpi); (**d**) and late stage of infection (9–30 dpi). Black lines between boxes indicate induction and red lines between boxes indicate suppression with dashed lines indicating a weak response and solid lines indicating a strong response. SA and JA synthesis gene designations are in black, PR protein gene designations are in red, TF gene designations are in blue, and ginsenoside synthesis gene designations are in purple. Arrows adjacent to gene designations indicate up or down gene regulation with bold indicating a stronger response. Illustration created with BioRender [18].

From these studies, it appears that increased JA-regulated ginsenoside synthesis is an important element of the defense response of ginseng plants against *Ilyonectria* spp. Early in the interaction, infection by the more aggressive *I. mors-panacis* triggered both JA and SA-synthesis gene expression, likely due to PAMP activity, but much more for SA synthesis gene expression, which corresponded to increased SA content suppressing the JA response, including TFs and ginsenoside biosynthesis-regulated genes (Figure 2a). Later in the interaction, this continued with even greater suppression of TFs and ginsenoside biosynthesis-regulated genes, and ginsenoside content only slightly increased (Figure 2b). In contrast, early in the interaction, infection by less aggressive *Ilyonectria* spp. did not significantly increase SA content and only slightly induced SA synthesis gene expression but not JA synthesis gene expression, and ginsenoside synthesis genes were induced along with ginsenoside accumulation (Figure 2c). Later in the interaction with the less aggressive *Ilyonectria* sp., there was induced SA, JA and ginsenoside synthesis gene expression and much greater ginsenoside accumulation (Figure 2d).

## 4. Pathogenesis-Related Proteins

PAMPs or effectors of plant pathogens typically trigger the production of host pathogenesis-related proteins (PR proteins) [49], which includes antioomycete and antifungal compounds, such as thaumatin-like proteins, chitinase and glucanase fungal cell wall degradation enzymes, proteinase inhibitors, and ribonucleases [50]. Among the DEGs identified in *P. ginseng* roots infected by *C. destructans* [29], there were 29 DEGs for defence response, which included genes for the PR proteins, *c55244_g1* described as part of the Bev v 1 family, and *c58299_g4* without a description. Expression of *c55244_g1* decreased from 0 to 0.5 dpi, increased at 4 dpi and decreased again at 7 dpi. Expression of *c58299_g4* increased from 0 to 0.25 dpi, decreased at 0.5 dpi, increased at 4 dpi and decreased again at 7 dpi. The authors concluded that 0.5 dpi was the critical time point for resistance given the peak in up-regulation of total DEGs and RGs, and thus the increase in expression of *c58299_g4* at 0.25 dpi could be part of the defence response. However, the fluctuating pattern of expression over time perhaps indicates periods of induction and suppression by PAMPs and effectors, respectively, or a relationship to other factors.

Polygalacturonase inhibiting proteins (PGIPs) are plant cell wall glycoproteins that are considered PR-like proteins, with leucine-rich repeats that bind and inhibit pathogen secreted pectinases and act in defence signalling [51]. Infection by *C. destructans* increased *PgPGIP* expression by 1-fold at 6 h post-infection (hpi) and 10.5-fold at 1 dpi, followed by a decrease to basal levels at 2 dpi and 3 dpi [32]. It is possible that *PgPGIP* was part of PTI triggered early in the infection and then later suppressed by effectors from *C. destructans*. The authors only referred to the pathogen as *C. destructans*, and so it is not possible to know whether a more or less aggressive *Ilyonectria* species was used.

The expression of two SA-responsive PR proteins, *PgPR2* encoding an acidic β-1,3-glucanase and *PgPR5* encoding a neutral thaumatin-like protein was compared between the infection by more or less aggressive *Ilyonectria* spp. in *P. ginseng* roots [28]. At 4 dpi with the more aggressive *I. mors-panacis*, *PgPR2* expression was 1-fold higher and *PgPR5* expression was 0.2-fold higher than the control, but by 16 dpi, *PgPR2* expression was 0.2-fold and *PgPR5* expression was 0.8-fold lower than the control. With the less aggressive *I. robusta,* however, expression was induced by 2.5-fold for *PgPR2* and 1-fold for *PgPR5* compared to the control at 4 dpi, while at 16 dpi, expression was induced 0.1-fold for *PgPR2* and 1-fold for *PgPR5* compared to the control. Although at 4 and 16 dpi, SA concentration was significantly greater than the control only for *I. mors-panacis*, the pathogen less induced expression of *PgPR2* and *PgPR5* than *I. robusta*, indicating that *I. mors-panacis* prevented downstream defence signalling for SA-related PR protein production, possibly by PTI-suppressive effectors, whereas *I. robusta* was unable to do that.

For *P. quinquefolius*, infection by *I. mors-panacis* caused expression of the JA-responsive *PqPR5,* encoding a neutral thaumatin-like protein, to significantly increase 10-fold at 1 dpi, remaining similar at 6 dpi and then significantly decreasing at 12 dpi [33]. Moreover, expression of the JA-responsive *PqChi-1* encoding a basic chitinase, significantly increased 4-fold at 1 dpi, a further 1.75-fold at 6 dpi and then remained unchanged at 12 dpi, and expression of the JA-responsive *PqPR10-2*, encoding a neutral ribonuclease, significantly increased 1.5-fold at 1 dpi and remained similar at 6 and 12 dpi. Expression of the SA-responsive PR protein, *PqGlu-1* encoding an acidic β-1,3-glucanase, significantly increased 3-fold at 1 dpi and then a further 1.6-fold at 12 dpi. Thus, both JA and SA-regulated PR protein expression were triggered early in the infection with SA-regulated PR protein gene expression further increasing late in the infection.

Based on these studies, the more aggressive *I. mors-panacis* induced SA accumulation early in the interaction with increased SA- and JA-related PR gene expression (Figure 2a). Later in the interaction, there were greater SA levels but with reduced expression of most SA-related PR gene expression indicating downstream suppression, possibly by effectors (Figure 2b). Less aggressive *Ilyonectria* spp. also induced SA-related PR protein gene expression early in the interaction, even though SA levels were not significantly increased (Figure 2c). Although SA levels were still not significantly increased later in the interaction with less aggressive *Ilyonectria* species, increased SA-related PR protein gene expression persisted (Figure 2d). As some of the previous studies only refer to the pathogen as *C. destructans* [29,32], a comparison between more and less aggressive species in their studies is not possible.

## 5. Altered Root Morphology

Invasion by plant pathogenic fungi can trigger morphological changes in the host, such as plant cell wall strengthening related to lignin deposition or wound-periderm formation [52]. An examination of structural changes in the cell wall of *P. ginseng* roots infected with a high and weakly virulent *C. destructans* isolate showed that only the highly virulent isolate produced black and soft necrotic lesions reaching the cortex of the roots by 7 dpi [31]. The weakly virulent isolate only caused yellow and dry lesions that were superficial with necrotic tissue restricted to the infection site. Infection by the weakly virulent isolate was associated with new layers of cell wall at 6 dpi with the number of layers expanding up to 12 dpi. At 20 dpi, there was complete separation of the infection site due to the layers of cell wall thickening, with an eventual detachment of the infected area. Based on this, it was concluded that the cell wall thickening layer was a wound periderm or an abscission layer, preventing further invasion by the weakly virulent *C. destructans* or damage from its enzymes and other secretion products [31]. The authors proposed that black root rot symptoms are caused by highly virulent *C. destructans* isolates, whereas rusty root symptoms are caused by weakly virulent *C. destructans* isolates. It is possible that highly virulent isolates are able to suppress cell wall strengthening with effectors enabling them to spread past the wound-periderm, but weakly virulent isolates lack those effectors, thus being contained by the wound-periderm. However, it is unknown if the highly and weakly virulent isolates were different species of *Ilyonectria* as the authors only referred to the pathogen as *C. destructans.*

Another study found that *P. ginseng* roots infected by *C. destructans* showed yellowish and dry superficial lesions at 1–4 dpi, but at 7 dpi, lesions became necrotic, with thickened cell walls partially disintegrated, damaged organelles, and intercellular hyphae reaching the xylem [30]. It was concluded that *P. ginseng* might have triggered defence responses at 4 dpi that became ineffective at 7 dpi. Necrotic tissue and the eventual death of the plant were attributed to the blockage of the xylem, restraining the adsorption of water and inorganic minerals. As the authors only referred to the pathogen as *C. destructans,* it is not possible to know which *Ilyonectria* species from this complex was used.

**Figure 3:**
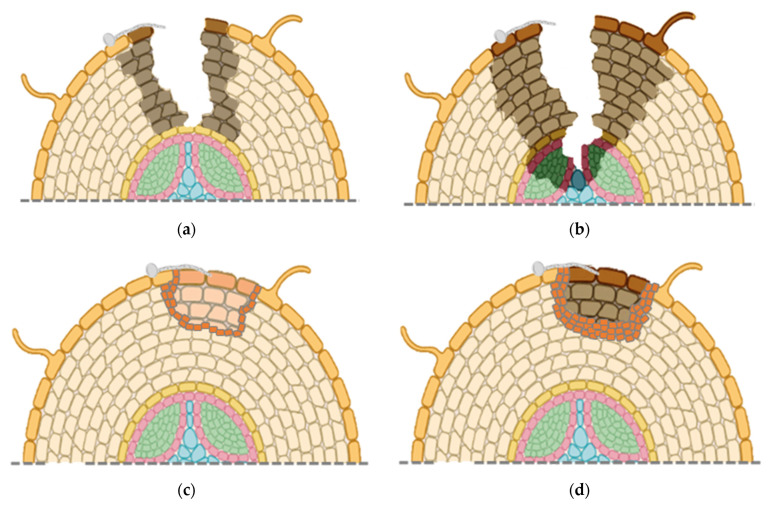
Ginseng root morphology with infection by the more aggressive *I. mors-panacis* (**a**) at the early (0.5–8 dpi); (**b**) or late stage of infection (9–30 dpi); (**c**) infection by less aggressive *Ilyonectria* spp. at the early (0.5–8 dpi); or (**d**) late stage of infection (9–30 dpi). The early stage of infection shows rot reaching the cortex with the more aggressive infection versus superficial rot with new cell layers and reinforced cell walls with the less aggressive infection. Late stage of infection shows rot reaching the xylem with more aggressive infection versus minimal rot with a well-developed wound periderm and/or abscission layer. Illustration created with BioRender [18].

Overall, changes in root morphology indicate that more aggressive isolates of *C. destructans*, possibly *I. mors-panacis*, first cause superficial infections (Figure 3a), but then can rapidly penetrate rotting the root cortex and xylem (Figure 3b). Weakly aggressive isolates of *C. destructans*, possibly other *Ilyonectria* spp., only colonize near the root surface with host cell wall strengthening early in the interaction (Figure 3c). Later in the interaction, there is separation of infected host tissue, possibly due to an abscission layer (Figure 3d).

## 6. Virulence Factors

Fungal plant pathogens can suppress PTI through secretion of effectors, which has been described as effector-triggered susceptibility (ETS) [53]. ETS by *I. mors-panacis* is supported by a decline in expression of the JA-regulated *PgPR5* gene at 12 dpi from a peak at 6 dpi [33], 2 SA-responsive PR protein genes at 16 dpi from a peak at 4 dpi [28], 13 ET-regulated DEGs and 7 JA-regulated at 12 dpi from a peak at 0.5 dpi [29], and 7 PRRs DEGs, 35 ET-regulated DEGs and 202 components of PTI/ETI DEGs at 12 dpi from a peak at 1.5 dpi [42]. Thus, results from several studies indicate that induction of many different types of ginseng defense genes early in the interaction (0.5–6 dpi) is followed by a later decrease in expression (12–16 dpi) implying early triggering of PTI followed by ETS.

There is some evidence for effectors produced by *I. mors-panacis*. Fungal small-secreted proteins (SSPs) have been showed to play a role as effectors suppressing the defense response and altering the physiology of their hosts [54]. Analysis of the secretome of *I. mors-panacis* found that 14.9% was composed of small-secreted proteins non-cysteine rich (SSNPs), and 7.9% was composed of small-secreted cysteine-rich proteins (SSCPs) [33]. Among the SSCPs, one showed homology to the *CRX1* effector for virulence by *Fusarium oxysporum* f. sp. *cepae* and another to the xylem 6 effector (*SIX6*) for virulence by *F. oxysporum* f. sp. *lycopersici*. There were 121 potential effectors genes in the secretome of *I. robusta* based on matches to the PHI database [42]. Among 20 genes examined, three were significantly up-regulated at 1.5, 3 and 6 dpi, another three were significantly up-regulated just at 3 and 6 dpi, and another four were significantly up-regulated just at 6 dpi. Thus, not all the potential effectors in the fungal genome were expressed during infection, and those that were expressed had different timings of induction indicating that they could have different targets in the host.

Ginsenoside saponins appear to be part of the defense response against *Cylindrocarpon*/*Ilyonectria* spp. [27,45]. However, the fungus can respond to ginsenoside exposure as 93 *I. mors-panacis* genes were found to be highly expressed when the fungus was cultured with *P. notoginseng* ginsenosides [34]. These included transcription factors, transporters, glycoside hydrolases and oxidoreductases, which might be responsible for regulation, transportation and detoxification of ginseng saponins. However, the role of the particular genes in virulence was not determined.

An examination of 22 isolates of several *Ilyonectria* species showed that four species that were avirulent to ginseng had similar metabolic profiles with each other, but they differed from those of *I. mors-panacis* and *I. robusta*, which had similar profiles and were virulent to ginseng [55]. The virulent species to ginseng produced the resorcylic acid lactone (RAL), radicicol. There were eight RALs isolated from an *I. mors-panacis* strain, which had antimicrobial activity and caused phytotoxicity to duckweed [56]. They noted that similar polyketides have been identified as virulence factors in several fungal plant pathogens suppressing basal plant defenses with RALs acting as strong inhibitors of plant Hsp90 chaperones negatively affecting defense responses, including reducing PTI. The authors proposed that RALs of *I. mors-panacis* may promote its virulence by suppressing ginseng defense responses.

Pathogens and their host compete for iron during infection with iron being involved in plant defense responses, and plants can withhold iron to limit the aggressiveness of pathogens [57]. However, plant pathogens can act against those defenses by secreting iron-scavenging siderophores to increase iron uptake and decrease iron-regulated host defense responses. Examples of the importance of siderophores for plant pathogens are the compromised virulence in siderophore deficient *Alternaria alternata* on citrus [58] and *Erwinia amylovora* on apple [59]. A siderophore N, N′, N″-triacetylfusarinine C (TAFC) was found to be only present in *Ilyonectria* species that were pathogenic to ginseng [60]. Based on the same siderophore in *Aspergillus fumigatus*, it was believed that iron was released from the siderophore by an esterase once taken up inside the cell. To test this, an *A. fumigatus* esterase that acts on TAFC was applied to ginseng roots inoculated with *I. mors-panacis*, and the esterase protected the roots from infection, supporting a role of this siderophore in *I. mors-panacis* virulence to ginseng [61].

As root rot is associated with pectolytic enzymes [62] and lesion browning is associated with phenol oxidation [63], an examination was undertaken into the production of plant cell wall degrading and phenol oxidizing enzymes as virulence factors of *C. destructans* against *P. quinquefolius* [20]. Highly virulent isolates differed from weakly virulent isolates by being able to directly penetrate the root epidermis, growing mostly intercellularly, with apparent degradation of the plant cell wall. At 14 dpi in wounded roots, highly virulent isolates created larger sunken dark brown lesions, whereas weakly virulent isolates created smaller sunken brown lesions. In culture, highly virulent isolates produced 1.8 units mg^−1^ dry mycelial weight^−1^ of pectinase, while weakly virulent isolates produced no detectable levels. Moreover, in culture, highly virulent isolates produced 61 units mg^−1^ dry mycelial weight^−1^ of polyphenoloxidase, while weakly virulent isolates produced just 30 units mg^−1^ dry mycelial weight^−1^. It was concluded that sunken brown lesions correlated with the ability of highly virulent isolates to produce higher levels of pectinase and polyphenoloxidase in culture, thus indicating that pectinase and polyphenoloxidase are key factors for *C. destructans* virulence. However, there were no measurements of the enzymes in roots, and so it is unknown if the correlation with virulence in culture also occurred during infection. As the authors only referred to the pathogen as *C. destructans*, it is not possible to know whether the highly and weakly virulent isolates were different *Ilyonectria* species.

In summary, virulence of *Ilyonectria* species to ginseng appears at least to be related to the production of potential effectors that may down-regulate SA and JA-regulated PTI, production of cell-wall degrading enzymes to damage host cells, proteins to transport and detoxify saponins, polyketides to suppress defense response, and siderophores to scavenge host iron. In addition, highly aggressive *I. mors-panacis*, but not the weakly aggressive *I. robusta,* significantly induced production of SA and ROS after infection, although the mechanism was not investigated [28]. It possibly could be similar to certain necrotrophic plant pathogens that can produce SA analogues, such as 5-formylsalicylic acid, that can be perceived by plants similar to SA to trigger defence responses [64]. Whatever the mechanism, higher virulence may result from *I. mors-panacis* triggering SA thus suppressing JA-related defenses.

## 7. Conclusions

*Ilyonectria/Cylindrocarpon* spp. are important soil-borne pathogens of ginseng causing localized or widespread (disappearing) root rot. While both more and less aggressive species can cause root rot, the interaction differs with infections by the more aggressive *I. mors-panacis* being characterized by increased SA content and suppression of JA-regulated genes early in the interaction, followed later in the interaction by very limited induction of cell wall defenses, ginsenosides, SA and JA-regulated PR proteins and other defenses. In contrast, infection by the less aggressive *Ilyonectria* spp. does not increase SA levels early in the interaction allowing for an up-regulation of JA-regulated defenses with induced cell wall defenses, increased ginsenoside content, JA-regulated PR proteins and other defenses. This suggests that *I. mors-panacis* is better able to manipulate PTI by using host antagonism between SA and JA responses, while possibly also having effectors that can effectively suppress SA-regulated PTI. Several possible virulence mechanisms have been proposed for *Ilyonectria*/*Cylindrocarpon* spp. that could help to obtain nutrients inside the host, suppress and detoxify host defenses, as well as damage host tissues. However, none of those mechanisms have yet been confirmed, such as by testing the virulence of isolates following candidate gene disruption or silencing.

## Data Availability

Not applicable.
